# Interruptions of nurses' activities and patient safety: an integrative literature review^1^

**DOI:** 10.1590/0104-1169.0251.2539

**Published:** 2015

**Authors:** Cintia Monteiro, Ariane Ferreira Machado Avelar, Mavilde da Luz Gonçalves Pedreira

**Affiliations:** 2 Master's student, Escola Paulista de Enfermagem, Universidade Federal de São Paulo, São Paulo, SP, Brazil. RN, Instituto de Oncologia Pediátrica, São Paulo, SP, Brazil. Scholarship holder from Coordenação de Pessoal de Nível Superior (CAPES), Brasil; 3 PhD, Adjunct Professor, Escola Paulista de Enfermagem, Universidade Federal de São Paulo, São Paulo, SP, Brazil; 4 PhD, Associate Professor, Escola Paulista de Enfermagem, Universidade Federal de São Paulo, São Paulo, SP, Brazil

**Keywords:** Nursing, Patient Safety, Human Engineering

## Abstract

**OBJECTIVES:**

to identify characteristics related to the interruption of nurses in professional practice, as well as to assess the implications of interruptions for patient safety.

**METHOD:**

integrative literature review. The following databases were searched: Pubmed/Medline, LILACS, SciELO and Cochrane Library, using the descriptors interruptions and patient safety. An initial date was not established, but the final date was December 31, 2013. A total of 29 papers met the inclusion criteria.

**RESULTS:**

all the papers included describe interruptions as a harmful factor for patient safety. Data analysis revealed three relevant categories: characteristics of interruptions, implications for patient safety, and interventions to minimize interruptions.

**CONCLUSION:**

interruptions favor the occurrence of errors in the health field. Therefore, there is a need for further studies to understand such a phenomenon and its effects on clinical practice.

## Introduction

Patient safety is a problem faced in the health field around the world. A study conducted in the United States of America (USA) identified the occurrence of adverse events during healthcare delivery as the 8th leading cause of death in the country^(^ 1 ^)^. A significant number of these adverse events is avoidable because they accrue from human errors of systemic origins^(^ 2 ^)^. Additionally, most of these errors occur due to the complexity of the care involved, considerable variation in the qualification and quantity of available healthcare providers, diversity of procedures, deficiencies in infrastructure and management, and mainly arise from failures in activity systems that disregard the human factor in the design and conception of actions^(^ 1 ^-^ 2 ^)^.

The nursing staff plays a key role in insuring the safety of patients because it provides direct assistance and care to the patient and family, composing the largest group of professionals in the health field in the world^(^ 3 ^)^.

Because these professionals have direct participation in the safety of patients, it is essential to understand the conditions and complexities of the working environment in which nurses work and that may compromise the quality of care delivery, especially in regard to interruptions of the activities performed by nurses.

According to the report To Err is Human: Building a Safer Health System, developed by the Institute of Medicine (IOM)^(^ 1 ^)^, interruptions contribute to the occurrence of errors and are the main cause of failures related to the work environment, very common in hospital facilities^(^ 4 ^-^ 5 ^)^.

Interruptions occur when the main task is suspended so that a secondary activity receives attention^(^ 6 ^)^. Interruptions can be classified into: intrusions (unexpected encounters with someone who temporarily interrupts the main activity), distractions (psychological responses triggered by external or environmental stimuli, or by secondary activities that break one's concentration on the primary task), breaks (planned or spontaneous pauses in a task), and disagreements (uncertainty perceived by the professional according to his/her knowledge, expectations and/or observations that are relevant for the work being performed)^(^ 7 ^)^.

Additionally, such interruptions can be a disturbing factor, affecting the professionals' concentration and delaying care delivery, impeding the professional from successfully finishing tasks, potentially favoring the occurrence of errors and putting patients at risk, in addition to wasting the resources of the healthcare system^(^ 8 ^)^. A task's cognitive load also influences the impact of interruptions on care delivery; human memory has limitations hindering the simultaneous assimilation of multiple inputs of information. 

Some interruptions are, however, essential in the process of care delivery and enable the transmission of important information^(^ 4 ^)^.

Nurses constantly perform multiple activities and need to develop cognitive mechanisms to keep their focus on clinical rationale, which is necessary to providing care. This dynamic environment in which tasks are performed requires reflection and complex psychomotor and cognitive skills to ensure quality and safe care delivery. Interruptions during practice may compromise the attention of workers, leading to distractions, and therefore, may represent a risk to the safety of patients. 

These distractions may be more related to failure in the systems than to individual performance^(^ 9 ^-^ 10 ^)^.

Note that patient safety is a result of the quality of interactions among all the components of the care system, not uniquely determined by one individual, type of activity, infrastructure or technology^(^ 11 ^)^. Therefore, to achieve good results it is essential to conceive and design environments and working processes in health and nursing, the fundamental principles of which are guided by the needs of patients and their families, comprising the causes and consequences of interruptions.

In the face of evidence that interruptions increase the likelihood of errors during care delivery and because of there being few international studies and no Brazilian studies characterizing such occurrences or describing their impact on clinical practice, this study's aim was to perform a literature review to understand the characteristics of interruptions and the factors contributing to this phenomenon, so as to implement strategies that enable reducing the occurrences of such events and improving quality of care.

Hence, this study's guiding question was defined as: "What are the interruptions experienced by nurses in their practice and how do these interruption compromise patient safety?"

## Objectives

This study's objective was to identify in the Brazilian and international literature characteristics related to the interruption of nurses in their professional practice and then assess the implications of such interruptions for patient safety.

## Method

This integrative literature review addresses the interruption of nurses, implications for patient safety and factors contributing to minimizing the occurrence of interruptions.

The purpose of this type of review is to synthesize a subject or theoretical framework to promote better understanding of an issue and to incorporate evidence into clinical practice. The stages of an integrative literature review include the identification of the topic and establishment of the research question; the establishment of inclusion and exclusion criteria of studies; the definition of information to be extracted from the selected studies; assessment of studies included in the review; interpretation of results and presentations of review^(^ 12 ^)^.

The descriptors used for the search were *interruptions and patient safety. *The following databases were included: Medical Literature Analysis and Retrieval System on Line (Medline), National Library of Medicine (Pubmed), Latin American and Caribbean Health Sciences (LiLACS), Scientific Electronic Library Online (SciELO) and Cochrane Library. An initial date was not established but the final date was December 31, 2013.

The inclusion criteria were: indexation in the previously identified databases; written either in English, Portuguese or Spanish; the study's objective should contain questions that indicated the topic was interruptions of nurses in clinical practice; and full-text articles. 

The exclusion criteria were papers addressing interruptions of activities developed but by healthcare providers other than nurses, book chapters, or letters to the readers.

First, we read the title of the publication followed by a careful reading of abstracts to verify whether the inclusion criteria were met. In cases in which the title and abstract were not sufficient to define the topic addressed, we sought the full-text so that all the criteria would be applied and papers answering the study's guiding question would be included.

The database search resulted in the identification of 290 papers. After applying inclusion and exclusion criteria, 29 (10%) papers were selected.

A form was developed to collect data to guide the reading and extraction of relevant data, which was filled in for each paper that was part of the final sample. Data were recorded concerning: the identification of papers and authors; year and country of publication; study's objectives; methodological characteristics; results; conclusions; and implications for nursing practice. 

The results and data analysis are presented in descriptive form.

## Results

All 29 papers assessed were published in periodicals published outside Brazil. In regard to databases, 19 (65.5%) papers were found both in PubMed and Medline, nine (31.0%) in PubMed, and one (3.5%) in Medline.

Among the 29 (100.0%) papers, 12 (41.4%) were conducted in the USA, five (17.2%) in Canada, four (13.8%) in Australia, two (7.0%) in Italy, two (7.0%) in the United Kingdom, one (3.4%) in China, one (3.4%) in Denmark, one (3.4%) in Germany, and one (3.4%) in Sweden.

In 13 (44.8%%) papers, the samples were exclusively composed of nurses (20.7%); six papers (20.7%) included the surgical staff (surgeon, anesthetist and nurse); three (10.4%) were composed of physicians and nurses and one (3.4%) paper verified a multidisciplinary team. Six (20.7%) papers were literature reviews.

All the papers included explored interruptions or mentioned them as being harmful to nurses' cognitive processes, leading to a greater number of errors, and consequently, compromising patient safety.

**Figure 1 -:**
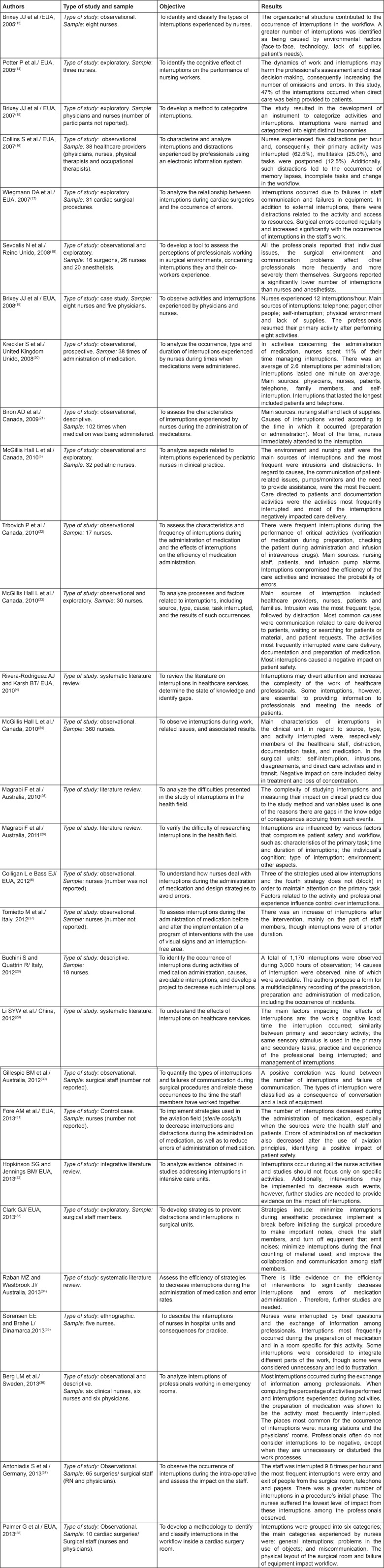
Presentation of papers according to the type, population, study's objective, main results and discussion.

Figure 1 presents the studies analyzed, which are presented according to author, methodological design, study sample and main results.

Analyzing the papers enabled the identification of three categories as the main aspects in the interruption of nurses in the routine of care delivery: characteristics of the interruption, which include frequency of occurrence, type, cause and source of interruption; activity interrupted; and place where the interruption occurred; implications of interruptions for patient safety; and interventions to minimize interruptions.

## Discussion

### Characteristics of interruptions

The number of interruptions experienced by nurses ranged from 0.4 to 13.9 interruptions per hour, according to the type of unit under observation^(^ 5 ^,^ 9 ^,^ 13 ^-^ 14 ^,^ 16 ^,^ 18 ^-^ 19 ^,^ 23 ^-^ 24 ^,^ 28 ^,^ 35 ^-^ 37 ^,^ 39 ^)^. Interruptions were most frequent in pediatric units, a fact that may be explained by the peculiar care environment of pediatric units due to the physiological characteristics and complex development of this population. It is also a dynamic unit with a high transit of family members, companions and workers^(^ 5 ^)^.

Additionally, the study showed that nurses are rarely able to complete an activity without being interrupted, which may be related to the tasks they constantly perform involving managing the unit, care delivery, and care directly provided to patients, in addition to being the most requested professional to provide information to patients, families and other healthcare providers(39).

Interruptions were more frequent when care was directly provided to patients, during the administration of medication, and completing documentation^(^ 4 ^-^ 5 ^,^ 14 ^,^ 23 ^-^ 24 ^,^ 35 ^-^ 36 ^)^. Some studies specifically assessed activities involving the administration of medication^(^ 6 ^,^ 20 ^-^ 22 ^,^ 27 ^-^ 28 ^,^ 31 ^,^ 34 ^)^, while others focused on interruptions during surgical procedures^(^ 17 ^-^ 18 ^,^ 30 ^,^ 33 ^,^ 37 ^-^ 38 ^)^.

Among the professionals in the surgical staff, surgeons are the professionals most frequently interrupted, followed by nurses^(^ 30 ^,^ 37 ^)^. One study, however, assessed the perceptions of professionals concerning this phenomenon and verified that surgeons reported being interrupted significantly less frequently than did nurses or anesthetists^(^ 18 ^)^. Additionally, there is a positive and significant linear correlation between interruptions in the workflow of surgical procedures and the occurrence of errors (p<0.001)^(^ 17 ^)^.

Only three studies classified interruptions according to type, while the most frequent interruptions resulted from intrusion and distraction, and less frequently, from disagreements and breaks^(^ 5 ^,^ 23 ^-^ 24 ^)^.

The main sources of the interruption of nurses were other healthcare providers, members of the nursing staff, telephones, pagers, patients, family members, visitors, and self-interruption^(^ 5 ^,^ 13 ^-^ 14 ^,^ 18 ^,^ 20 ^,^ 22 ^-^ 24 ^,^ 27 ^,^ 30 ^,^ 35 ^,^ 37 ^)^. There are also the environment's physical characteristics^(5,13,38) ^and a lack of supplies or a failure of equipment necessary for care delivery^(^ 13 ^,^ 21 ^,^ 30 ^,^ 38 ^)^, which lead to interruptions in the workflow. Researchers report that nurses were more frequently interrupted to answer questions concerning professional issues and due to the need to provide patient-related information^(^ 35 ^)^. In regard to places where interruptions occur, research shows there is greater occurrence in nursing stations, followed by rooms dedicated to storing and preparing medication , medical staff rooms, areas near beds and corridors^(^ 35 ^-^ 36 ^)^.

One study reports that one nurse was interrupted 43 times in a period of 10 hours; 23% of these occurrences accrued from operational failures such as lack of supplies, equipment or personnel^(^ 40 ^)^. These interruptions caused by failures in the system are avoidable, therefore, working processes in healthcare facilities should be improved to minimize such occurrences. Where these failures are corrected, nurses spend less time resolving institutional failures and have more time to provide direct care to patients^(^ 41 ^)^.

A characteristic observed in one study assessing interruptions during the administration of medication was that among the 14 causes of interruptions, nine (64.3%) were avoidable. The most frequent reasons were: illegible or incomplete medical prescriptions; the need to address the requests of physicians or other providers; and alarms. All of these are avoidable interruptions^(^ 28 ^)^.

An instrument called the "Hybrid Method to Categorize Interruptions and Activities" (HyMCIA) was developed to allow professionals to understand the activities performed and interruptions in the workflow. This method classified activities using Grounded Theory and simultaneously developed a hybrid method to classify interruptions. The analysis of these observations resulted in the development of a taxonomy of interruptions and a chronology of activities and interruptions, which increased the likelihood of understanding discontinuities in workflow caused by interruptions^(^ 15 ^)^.

The interruptions were categorized into: recipient - the person who was interrupted; unintended recipient - the person was not intended to be interrupted; indirect recipient - person who was indirectly affected by an interruption; self-interruption - the worker him/herself interrupted his/her task without the intervention of another person; distraction - interruption caused by lack of attention; organizational structure - interruption caused by failures in the work area's physical structure; lack of supplies - interruption originated from a need to seek materials or equipment not available in the work area; and initiator - the person who caused the interruption^(^ 15 ^)^.

### Implications of Interruptions for Patient Safety

Interruptions directly affect the performance of activities and may compromise decision-making processes and the efficiency of workers when they occur during the performance of more complex activities that require greater concentration^(^ 7 ^)^. These occurrences are common in the practice of nurses and impact the quality and safety of care delivered to patients by interfering in staff member's cognitive processes, potentially resulting in a great number of errors^(^ 4 ^-^ 6 ^,^ 13 ^-^ 14 ^,^ 16 ^-^ 17 ^,^ 22 ^-^ 24 ^,^ 26 ^-^ 27 ^,^ 29 ^-^ 30 ^,^ 35 ^)^. Additionally, one study reports that interruptions unnecessary for care generate frustration, stress, and demotivate professionals^(^ 35 ^-^ 36 ^)^.

Studies identified that 88.9% to 90% of interruptions resulted in negative consequences, such as delay in treatment and loss of concentration^(^ 5 ^,^ 23 ^-^ 24 ^)^. Other studies related interruptions to a greater chance of errors in the administration of medication^(^ 6 ^,^ 27 ^)^.

The literature shows that interruptions do not always lead to adverse events and some may have a positive impact on a professional's performance and care delivery because some interruptions may contribute to increased safety, increased comfort of patients, and help nurses to be more accurate in their tasks^(^ 5 ^)^.

Therefore, further studies addressing this topic with methodology appropriate to the study's objective^(25-26) ^are needed to assess the impact of interruptions on care delivery, since some interruptions are actually necessary to quality care^(^ 4 ^-^ 5 ^,^ 32 ^,^ 35 ^-^ 36 ^)^.

### Interventions to Minimize Interruptions

Data analysis shows the important need to improve and restructure the health system with the goal to manage and minimize the number of harmful interruptions, thus ensuring patient safety and the quality of nurses' work.

The identification of conditions that cause interruptions in the work of nurses may contribute to the development of strategies to avoid this occurrence and minimize the impact on care delivery. These interventions, however, are more efficient when they involve and sensitize the entire staff in regard to the great probability of posing risks to patients.

Ten studies addressed strategies of interventions, including the management of processes, activities-support tools, signalization of interruption-free areas, and continuous education of the staff to qualify both those being interrupted and those who are doing the interrupting, controlling interruptions, and considering the priorities and times with a greater risk of harming the work process and patient safety^(^ 4 ^,^ 6 ^,^ 20 ^,^ 24 ^,^ 27 ^-^ 29 ^,^ 31 ^,^ 33 ^-^ 34 ^)^.

Other important factors that enable putting into practice changes that lead to a smaller number of interruptions involve education, motivation and cooperation within the team; the commitment and interest of managers; an appropriate number of professionals and collaboration among them; decreased overload; and the modification of behavior of other healthcare providers, patients and/or family members^(^ 4 ^,^ 6 ^,^ 24 ^,^ 28 ^,^ 33 ^)^.

The intervention designed to decrease the number of interruptions during the administration of medications established: an area exclusively dedicated to prepare medications; use by the nurse responsible for administering medications of a red vest with the following words on it "Please, do not interrupt, I am administering medications"; and the use of educational strategies^(^ 27 ^)^. After such interventions, however, an increased number of interruptions were observed, especially during the time when medication was being prepared, mainly by staff members. Nonetheless, interruptions were or shorter duration and the time nurses dedicated to the performance of direct care increased, enabling the supply of care to a greater number of patients^(^ 27 ^)^. Another study, however, which implemented strategies used in the aviation field (sterile cockpit), showed a decreased number of interruptions during the administration of medication, especially when the sources were the health staff and patients^(^ 31 ^)^.

Interruptions, however, may still occur, even after instructing the staff and adopting strategies to decrease the number of interruptions, depending on patient needs and staff adherence to recommendations.

Therefore, it is necessary for nurses to be able to deal with the occurrence of interruptions. One study aiming to understand how nurses manage interruptions during the administration of medications reports four strategies. Three of these allow interruptions: when the primary task is discontinued and can be resumed after a secondary task that has greater priority is performed; when the professional shares attention between the primary and secondary tasks, as both have similar priority; and when an interruption is mediated with an action that allows the primary task to be resumed (prospective memory). The fourth intervention, however, called "blocking", occurs when the primary task has greater importance and the interruption must be blocked, so that the professional is able to maintain attention to this primary task. Note that these strategies depend on staff workload and clinical assessments and are influenced by factors related to the activities involved and professional experience^(^ 6 ^)^.

## Conclusion

The occurrence of interruptions is a constant in the environment of healthcare delivery because it involves patients with different levels of complexity and dynamics of healthcare delivery, in addition to interactions with various healthcare providers and sectors.

This review enabled the identification of relevant aspects in nursing practice that favor the occurrence of interruptions. Nonetheless, few studies describe the impact of interruptions for clinical practice and patient safety and most papers only describe the characteristics of interruptions and present few proposals of interventions to implement them into practice. 

Therefore, further studies are needed to identify environmental and human factors that contribute to the occurrence of interruptions, to the assessment of the impact of interruptions on care, the design of the work system, and the design of easy-to-implement and efficient strategies to support nurses in better managing interruptions in a complex and dynamic working environment.

## Final considerations

Studies presented in this review show a scarcity of papers addressing interruptions during the practice of nurses, which may be related to the absence of a descriptor for this topic that is used worldwide. Additionally, no study addressing this topic was found in Brazil, which hinders comparisons with the Brazilian context in which nurses often have to correct failures in the system and the nursing staff is mostly composed of professionals without a bachelor's degree.
